# Interleukin-6/soluble IL-6 receptor-induced secretion of cathepsin B and L from human gingival fibroblasts is regulated by caveolin-1 and ERK1/2 pathways

**DOI:** 10.3389/fdmed.2025.1547222

**Published:** 2025-03-11

**Authors:** Ayaka Goto, Kazuhiro Omori, Tomoko Yamaguchi-Tomikawa, Hiroya Kobayashi, Yuki Shinoda-Ito, Kimito Hirai, Atsushi Ikeda, Shogo Takashiba

**Affiliations:** ^1^ Department of Pathophysiology-Periodontal Science, Graduate School of Medicine, Dentistry and Pharmaceutical Sciences, Okayama University, Okayama, Japan; ^2^ Department of Periodontics & Endodontics, Division of Dentistry, Okayama University Hospital, Okayama, Japan

**Keywords:** cathepsin B, cathepsin L, human gingival fibroblast, interleukin-6, caveolin

## Abstract

**Aims:**

Cathepsins are essential lysosomal enzymes that maintain organismal homeostasis by degrading extracellular substrates. The inflammatory cytokine interleukin-6 (IL-6) increases the production of cathepsins through the caveolin-1 (Cav-1) and c-Jun N-terminal kinase (JNK) signaling pathways, which have been implicated in the destruction of periodontal tissue. This study investigated the effect of the IL-6/soluble IL-6 receptor (sIL-6R) complex on the extracellular secretion of cathepsins in human gingival fibroblasts (HGFs) and examined the function of extracellularly secreted cathepsins B and L under acidic culture conditions *in vitro*.

**Methods:**

HGFs were isolated from healthy volunteer donors. The expression of Cav-1 was suppressed via transfection with small interfering RNA (siRNA) targeting Cav-1. The expression levels of cathepsins B and L induced by extracellular IL-6/sIL-6R were measured using western blotting and enzyme-linked immunosorbent assay. Extracellular cathepsin activity following IL-6/sIL-6R stimulation was assessed using a methylcoumarylamide substrate in a fluorescence-based assay. IL-6/sIL-6R-induced expression of cathepsins B and L in HGFs was quantified under inhibitory conditions for extracellular signal-regulated kinase (ERK) 1/2 and/or JNK signaling, both of which are transduction pathways activated by IL-6/sIL-6R. This quantification was also performed in HGFs with suppressed Cav-1 expression using western blotting.

**Results:**

Cathepsins B and L were secreted in their precursor forms from HGFs, with significantly elevated protein levels observed at 24, 48, and 72 h post-IL-6/sIL-6R stimulation. Under acidic culture conditions, cathepsin B activity increased at 48 and 72 h. Cav-1 suppression inhibited the secretion of cathepsin B regardless of IL-6/sIL-6R stimulation, whereas the secretion of cathepsin L was reduced only after 48 h of IL-6/sIL-6R stimulation. Inhibition of ERK1/2 and JNK pathways decreased the secretion of cathepsin B after 48 h of IL-6/sIL-6R stimulation, and JNK inhibition reduced the secretion of cathepsin L under similar conditions.

**Conclusion:**

IL-6/sIL-6R stimulation increased the extracellular secretion of cathepsin B and L precursors in HGFs, and these precursors became activated under acidic conditions. Cav-1 and ERK1/2 are involved in regulating the secretion of cathepsin B precursors.

## 1 Introduction

Lysosomes are organelles found in human cells that function in acidic environments. They contain various hydrolytic enzymes essential for maintaining homeostasis by breaking down unnecessary proteins and metabolites into amino acids (1). Cathepsins, one of the most abundant proteases in lysosomes, are widely distributed across organisms and play a key role in degrading extracellular matrix components such as collagen, elastin, and laminin (2). The cathepsin family consists of 15 types of lysosomal proteases (3, 4) present in nearly all organisms to maintain homeostasis by degrading extracellular proteins. Based on amino acid sequences, at least 11 types of cathepsins have been identified and classified as cysteine or serine proteases (5, 6). The distribution of cathepsins varies depending on their type and the tissue in which they are found. For example, cathepsins B, L, and H are present in nearly all human cells, whereas cathepsin K is predominantly found in osteoclasts (7).

The process of cathepsin production and activation involves several steps: (1) synthesis of cathepsin precursors in the endoplasmic reticulum, (2) transport to the Golgi apparatus, (3) addition of mannose 6-phosphate, (4) transport to the lysosomes, and (5) N-terminal cleavage under acidic pH conditions (8). Previous studies have linked mutations in cathepsin genes to various diseases. For instance, pycnodysostosis is characterized by osteosclerosis and short stature and results from a deletion in the cathepsin K gene (9). Similarly, Papillon-Lefèvre syndrome, marked by palmoplantar hyperkeratosis and severe early-onset periodontitis, is caused by a deletion in the cathepsin C gene (10, 11). In addition, cathepsin L deficiency has been closely associated with gingival hyperplasia in mouse models (12).

Interleukin-6 (IL-6) is a cytokine involved in the immune and hematological systems, playing a critical role in biological defense by promoting B-cell differentiation and T-cell activation (13–15). It is also implicated in various inflammatory diseases such as rheumatoid arthritis and periodontitis (16, 17). IL-6 binds to the membrane-bound IL-6 receptor (mIL-6R) and activates cell signaling by engaging the membrane-bound glycoprotein 130 (gp130) (18). However, in cells lacking mIL-6R, such as human gingival fibroblasts (HGFs), IL-6 binds to the soluble IL-6 receptor (sIL-6R) in the serum, forming a complex that then interacts with gp130 to transmit signals into the cell (19, 20).

IL-6, induced by periodontal bacteria, plays a key role in the development of periodontitis (21). The IL-6/sIL-6R complex regulates the production of several proteins such as matrix metalloproteinases (MMPs) and vascular endothelial growth factor (VEGF) in HGFs, leading to connective tissue degradation and angiogenesis, and ultimately resulting in periodontal tissue destruction (22–24). The IL-6/sIL-6R complex also increases the production and activity of cathepsins B and L, two enzymes that contribute to connective tissue degradation. Signal transduction from the plasma membrane to the nucleus is mediated by caveolin-1 (Cav-1) and c-Jun N-terminal kinase (JNK) (25–27), which are major components of the cholesterol- and sphingolipid-rich curved lipid rafts in the plasma membrane. Cav-1 is widely present in the human body, represented by endothelial cells, smooth muscle cell cells, skeletal muscle cells, fibroblasts, type I alveolar cells, and adipocytes, and is associated with various functions such as endocytosis, extracellular matrix organization, cholesterol distribution, cell migration, and signal transduction. Therefore, it has been implicated in cancer development, inducing insulin resistance in diabetes, and in the progression of Alzheimer's disease, making it a potential target for disease prevention (28). Studies have shown that the expression of cathepsin B and L messenger RNA (mRNA) is elevated in the gingival fibroblasts of patients with periodontal disease (29). Therefore, IL-6 may contribute to periodontal tissue destruction through the cathepsin pathway, in addition to its effects on MMPs and VEGFs.

Cathepsin B is often found in the synovial fluid of patients with rheumatoid arthritis and has been linked to articular cartilage degradation (30). Moreover, increased cathepsin B expression has been observed in several malignancies, including breast, colorectal, and oral squamous cell cancers, where it is associated with tumor invasion, autophagy, angiogenesis, and metastasis (31, 32). Consequently, many studies have explored the role of cathepsins in chronic inflammatory lesions and tumors, both within and outside cells (33).

Our previous study demonstrated that IL-6, in the presence of sIL-6R, enhanced cathepsin B and L activity in human gingival fibroblasts (HGFs) via the Cav-1 signaling pathway (25). However, the mechanisms underlying the extracellular expression and functional activity of these cathepsins in the presence of IL-6 and sIL-6R remain unclear. In this study, we investigated the mechanisms regulating the extracellular secretion and activation of cathepsins B and L in HGFs following IL-6/sIL-6R stimulation. While our previous study identified Cav-1 as a key regulator of intracellular cathepsin production, its role in extracellular secretion remained unclear. Additionally, the involvement of other signaling pathways, such as ERK1/2, had not been explored.

We found that Cav-1 was essential not only for intracellular production but also for extracellular secretion of cathepsins. Furthermore, ERK1/2 and JNK pathways regulate cathepsin B secretion, whereas JNK specifically controls cathepsin L secretion, indicating distinct regulatory mechanisms. Finally, we confirmed that extracellularly secreted cathepsins become enzymatically active under acidic conditions. These findings expand our understanding of cathepsin regulation in periodontal inflammation and highlight Cav-1 and ERK1/2 as potential therapeutic targets for preventing periodontal tissue destruction.

## 2 Methods

### 2.1 Reagents

Recombinant human IL-6 and sIL-6R were purchased from R&D Systems (Minneapolis, MN, USA). Rabbit-derived polyclonal antibodies against human cathepsins B (Catalog number: sc-13985) and L (Catalog number: sc-10778), small interfering RNA (siRNA) targeting Cav-1, and scrambled control siRNA were purchased from Santa Cruz Biotechnology (Dallas, TX, USA). The extracellular signal-regulated kinase (ERK) 1/2 inhibitor PD98059 and JNK inhibitor SP600125 were purchased from Merck (Darmstadt, Germany). The 4-methyl-7-coumarylamide (MCA) peptide substrates, Z-Arg-Arg-MCA (Product Code number: 3123-v) and Z-Phe-Arg-MCA (Product Code number: 3095-v), were purchased from Peptide Institute (Osaka, Japan) and used to measure cathepsin activity.

IL-6 and sIL-6R were dissolved in phosphate-buffered saline (PBS; Thermo Fisher Scientific, Waltham, MA, USA) to a concentration of 50 µg/ml each and stored at −80°C. PD98059 and SP600125 were dissolved in dimethyl sulfoxide (DMSO; Merck) to a concentration of 50 mM each and stored at −80°C.

### 2.2 Isolation and culture of human cells

HGFs were isolated and cultured from healthy human gingival tissue following the method described by Naruishi et al. (20), with informed consent obtained from one donor. This study was approved by the Ethics Committee of Okayama University Graduate School of Medicine, Dentistry, and Pharmaceutical Sciences and Okayama University Hospital (Approval No. 661). The cells were cultured in Dulbecco's modified eagle medium (DMEM; Thermo Fisher Scientific) containing 20 mM 4-(2-hydroxyethyl)-1-piperazineethanesulfonic acid (HEPES; Merck), 10% fetal bovine serum (FBS; Biowest SAS, Nuaillé, France), and 1% penicillin-streptomycin (Thermo Fisher Scientific) at 37°C, 5% CO_2_, and 95% humidity. HGFs were subcultured at a 1:4 ratio once cell density reached 80% confluence, and five to eight passages were used for the experiment. Prior to the addition of IL-6 and sIL-6R, HGFs were cultured in DMEM with 20 mM HEPES, 0.5% FBS, and 1% penicillin-streptomycin (DMEM + 0.5% FBS) for 24 h to align the cell cycle with the quiescent phase.

### 2.3 Establishment of Cav-1 knockdown HGFs and stimulation of IL-6/sIL-6R complex

Knockdown of Cav-1 expression in HGFs was performed following the method described by Yamaguchi et al. (25). Briefly, HGFs were seeded in 35 mm dishes (Corning, New York, NY, USA) at a density of 5.0 × 10^4^ cells/cm^2^ and cultured as previously described. Cav-1 siRNA or negative control siRNA (100 nM), diluted in Opti-MEM (Thermo Fisher Scientific), was transfected using the Lipofectamine Transfection Reagent and Plus Reagent (Thermo Fisher Scientific). To minimize the cytotoxicity of Lipofectamine, the culture medium was added 5 h after the transfection reagent. The medium was then replaced with DMEM + 0.5% FBS 24 h after siRNA transfection, followed by the addition of a mixture of 50 ng/ml IL-6 and 50 ng/ml sIL-6R (IL-6/sIL-6R complex) 48 h later.

### 2.4 Effect of ERK1/2 and JNK inhibition on cathepsin secretion by IL-6/sIL-6R stimulation

HGFs were seeded in 35 mm dishes at a density of 5.0 × 10^4^ cells/cm^2^ and cultured as previously described. The transduction pathways of HGFs were subsequently inhibited with or without 50 µM of ERK1/2 inhibitor (PD985059) and JNK inhibitor (SP600125), followed by stimulation with the IL-6/sIL-6R complex (50 ng/ml each) after 30 min (24, 25).

### 2.5 Quantification of extracellular cathepsins B and L using western blotting

The effect of IL-6/sIL-6R complex stimulation on the secretion of cathepsins B and L in HGFs was examined using western blotting (34). The cells were seeded and cultured as previously described, and the culture supernatant was collected every 24 h after the addition of the IL-6/sIL-6R complex. The supernatant was then centrifuged at 9,500 × *g* for 10 min at 4°C to remove floating cells. The samples were stored at −80°C immediately after supernatant collection.

Protein assays were performed as described by Bradford et al. (35). A total of 5 µg of the collected samples were mixed with sodium dodecyl sulfate (SDS) sample buffer (45 mM Tris-HCl, pH 6.8, 15% glycerol, 1% SDS, and 144 mM β-mercaptoethanol) and boiled for 5 min to reduce proteins. All samples were kept on ice until fully reduced. The samples were separated using SDS-polyacrylamide gel electrophoresis with 12% acrylamide gels and electrophoresis buffer (25 mM Tris-HCl, 200 mM glycine, and 35 mM SDS) under a voltage of 150 V at room temperature. The separated proteins were subsequently transferred to a polyvinylidene difluoride (PVDF) membrane (Merck) using a wet transfer device (MINI PROTEAN® II; Bio-Rad Laboratories, Hercules, CA, USA) and transfer buffer (1.8 mM Tris-HCl, 190 mM glycine, and 20% methanol) under a voltage of 100 V for 60 min at 4°C. Blocking of the transferred PVDF membranes was performed using Tris-buffered saline (TBS; 10 mM Tris-HCl, 150 mM NaCl, pH 7.4) containing 5% skim milk (BD Biosciences, Franklin Lakes, NJ, USA) for 1 h at 4°C.

The PVDF membranes were shaken in a primary antibody solution diluted with 5% skim milk in TBS for 12 h at 4°C. The following antibodies were used: rabbit-derived anti-human cathepsin B polyclonal IgG antibody at a 1:250 dilution and rabbit-derived anti-human cathepsin L polyclonal IgG antibody at a 1:200 dilution. After washing with TBS containing 0.05% Tween-20 (T-TBS), the membranes were shaken in a secondary antibody solution diluted with 5% skim milk in TBS for 1 h at 4°C. The secondary antibody used was a horseradish peroxidase (HRP)-conjugated anti-rabbit IgG antibody (GE Healthcare UK Ltd, Buckinghamshire, United Kingdom) at a 1:1,000 dilution. Reactive proteins were detected using an enhanced chemiluminescence system (SuperSignal® West Dura Extended Duration Substrate; Thermo Fisher Scientific).

For reprobing, the membranes were shaken in an antibody removal buffer (Restore^TM^ Western Blot Stripping Buffer; Thermo Fisher Scientific) for 30 min at room temperature to remove the antibodies, allowing for the detection of different proteins on the same PVDF membranes with other antibodies. The same blocking and antibody reaction procedures were then repeated as previously described. The relative intensity of the bands corresponding to the target proteins was quantified based on the intensity at 0 h (without IL-6/sIL-6R complex stimulation) using the image analysis software Image J® (version 1.46r, NIH, Bethesda, MD, USA). The membranes were stained with Coomassie Brilliant Blue (CBB) solution to confirm equal protein loading in each lane.

### 2.6 Determination of extracellular cathepsin B precursors by enzyme-linked immunosorbent assay (ELISA)

The amount of cathepsin B precursors in the culture supernatant was quantified using the Human Pro-Cathepsin B Quantikine® ELISA Kit (R&D Systems). HGFs were seeded in 35 mm dishes at a density of 5.0 × 10^4^ cells/cm^2^ and cultured as previously described, followed by stimulation with the IL-6/sIL-6R complex. Culture supernatants were collected at 0, 24, 48, and 72 h after stimulation for measurement.

### 2.7 Measurement of extracellular cathepsin B and L activity

The activity levels of cathepsins B and L in the culture supernatant were measured using the fluorescent peptide MCA substrate (36). Supernatants were collected at 0, 24, and 48 h after IL-6/sIL-6R complex stimulation and centrifuged at 9,500 × *g* for 10 min at 4°C. Protein assays were then performed using the Bradford method (35). One microgram of the obtained sample was incubated with the substrate (Z-Arg-Arg-MCA for cathepsin B, and Z-Phe-Arg-MCA for cathepsin B + L) in a sodium acetate solution [4 mM ethylenediaminetetraacetic acid (EDTA) and 0.4 M sodium acetate] at pH 5.5 for 90 min at 37°C. The relative activities of free 7-amido-4-methylcoumarin (AMC) were quantified based on the intensity at 0 h after IL-6/sIL-6R complex stimulation, with fluorescence intensity detected at a 460 nm emission wavelength after excitation at 380 nm using a fluorescent microplate reader (Gemini XPS; Molecular Devices, San Jose, CA, USA).

### 2.8 Statistical analysis

Statistical analysis for all experiments was performed using one-way analysis of variance (ANOVA), followed by Scheffe's test or Fisher's protected least significant difference (PLSD) test for multiple comparisons, using SPSS software (version 13.0; Chicago, IL, USA). Statistical significance was indicated by *p*-values less than 0.05.

## 3 Results

### 3.1 IL-6/sIL-6R complex stimulation increases the secretion of cathepsin B and L precursors

**Figure 1:**
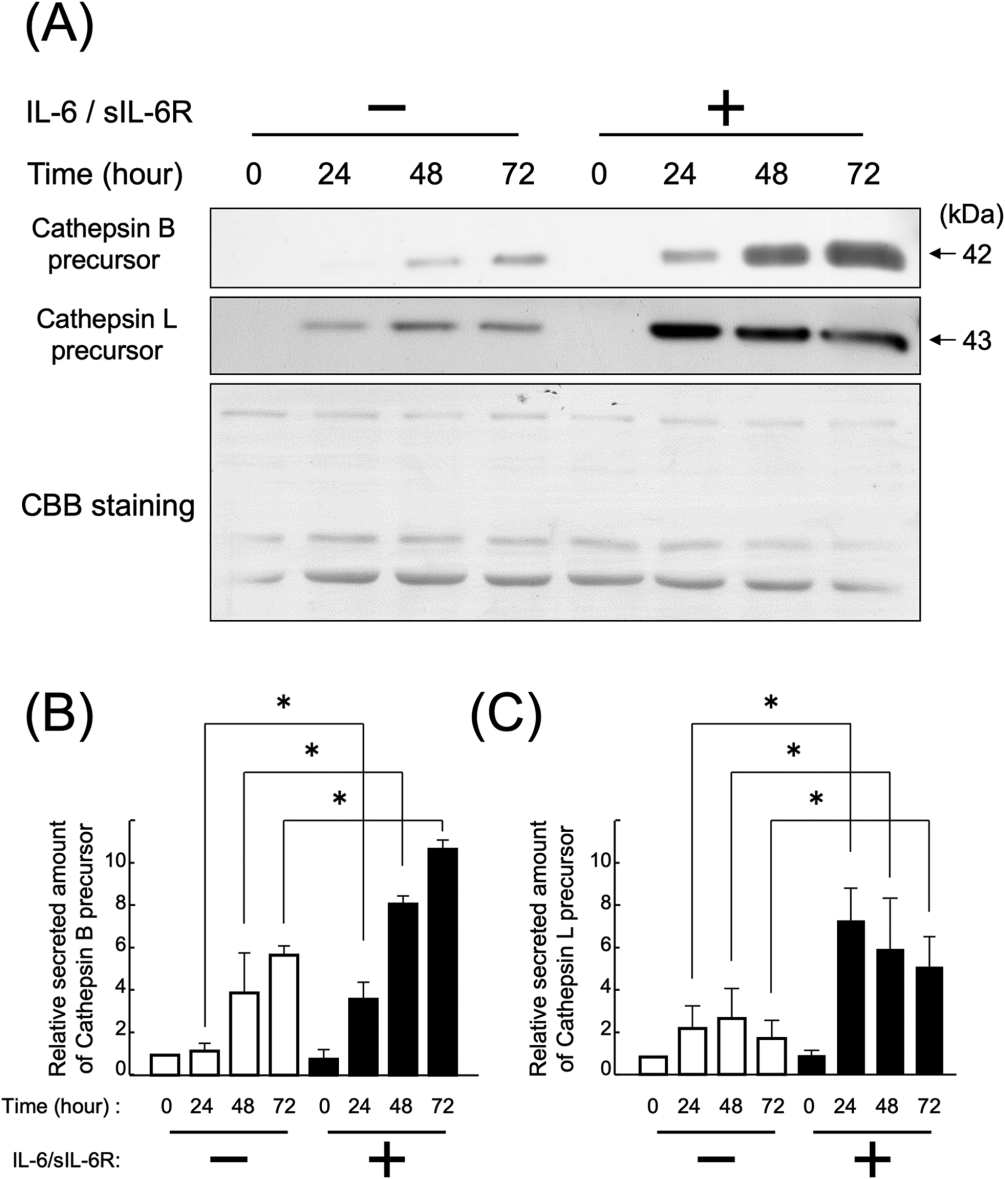
Effect of IL-6/sIL-6R complex stimulation on the secretion of cathepsin B and L precursors from HGFs. Cathepsin B and L precursors were detected in the culture supernatants of HGFs after stimulation with the IL-6/sIL-6R complex at 0, 24, 48, and 72 h using western blotting. HGFs were sourced from one donor, and three independent experiments were conducted. The intensity of the bands detected by western blotting was quantified using the ImageJ® software, with values calculated relative to the 0 h time point without IL-6/sIL-6R complex stimulation. The graph shows the mean ± SD, with error bars representing deviations. Statistical analyses were performed using one-way ANOVA and Scheffe's test. **(A)** Western blot images of cathepsin B and L precursors in the cell culture supernatant, with representative images displayed. CBB staining verified that protein amounts loaded in each lane were comparable. **(B)** Densitometric analysis of secreted amounts of cathepsin B precursors. The *y*-axis displays the relative densitometric values of the secreted cathepsin B precursors, whereas the *x*-axis shows the culture duration with or without IL-6/sIL-6R complex stimulation. *n* = 3; *: *p* < 0.05. **(C)** Densitometric analysis of secreted amounts of cathepsin L precursors. The *y*-axis displays the relative densitometric values of the secreted cathepsin L precursors, whereas the *x*-axis shows the culture duration with or without IL-6/sIL-6R complex stimulation. *n* = 3; *: *p* < 0.05. ANOVA, analysis of variance; CBB, Coomassie Brilliant Blue; HGFs, human gingival fibroblasts; IL-6, interleukin-6; SD, standard deviation; sIL-6R, soluble interleukin-6 receptor.

**Figure 2:**
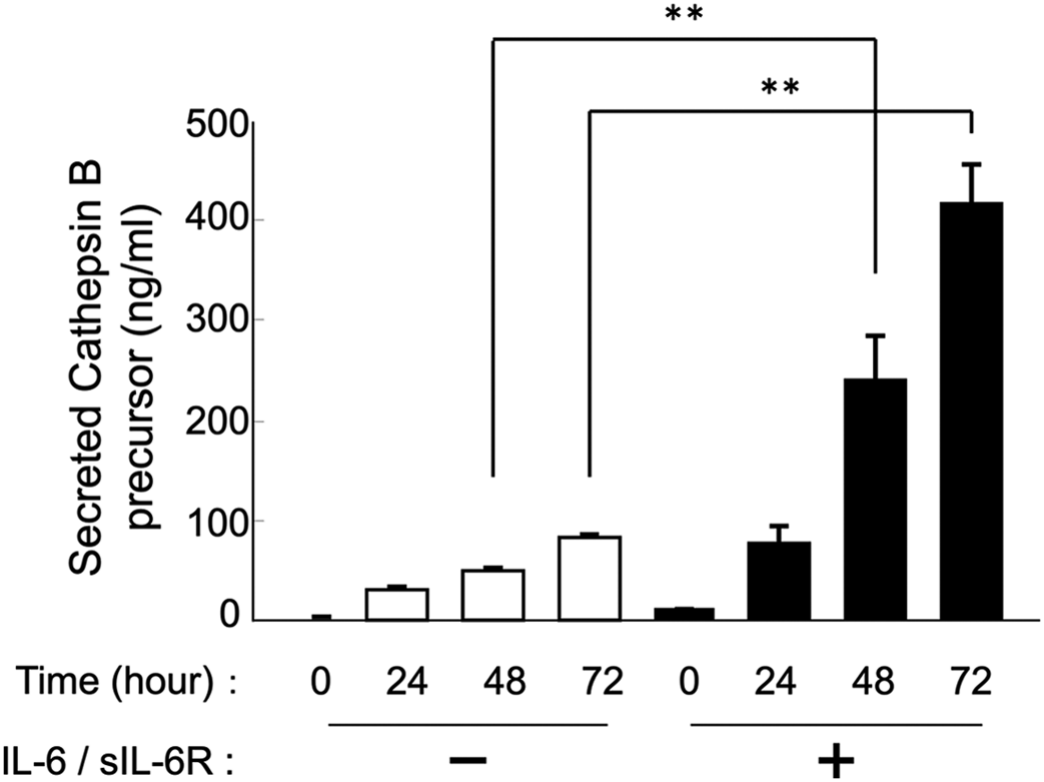
Effect of IL-6/sIL-6R complex stimulation on the secretion of cathepsin B precursors from HGFs. Cathepsin B precursor levels were measured in the culture supernatants of HGFs at 0, 24, 48, and 72 h after cell culture with IL-6/sIL-6R complex stimulation using ELISA. The *y*-axis represents the relative concentration of secreted cathepsin B precursors, whereas the *x*-axis denotes the culture duration with or without IL-6/sIL-6R complex stimulation. HGFs were sourced from one donor, and three independent experiments were conducted. The error bars in the graph represent the mean ± SD. Statistical analyses were performed using one-way ANOVA and Fisher's PLSD test. *n* = 3; **p < 0.01. ANOVA, analysis of variance; ELISA, enzyme-linked immunosorbent assay; HGFs, human gingival fibroblasts; IL-6, interleukin-6; PLSD, protected least significant difference; SD, standard deviation; sIL-6R, soluble interleukin-6 receptor.

HGFs secreted cathepsins B and L even without IL-6/sIL-6R complex stimulation (Figures 1, 2). Western blotting of the culture supernatant detected precursors of cathepsins B and L based on their molecular weights. The analysis revealed a significant increase in the secretion of cathepsin B and L precursors at 24, 48, and 72 h after IL-6/sIL-6R complex stimulation compared with the negative control (non-stimulation group) (Figure 1; *p* < 0.05). Similarly, ELISA showed an increase in the secretion of cathepsin B precursors at 48 and 72 h after IL-6/sIL-6R complex stimulation (Figure 2; *p* < 0.05).

### 3.2 IL-6/sIL-6R complex stimulation increases the activity of secreted cathepsin B and L precursors

**Figure 3:**
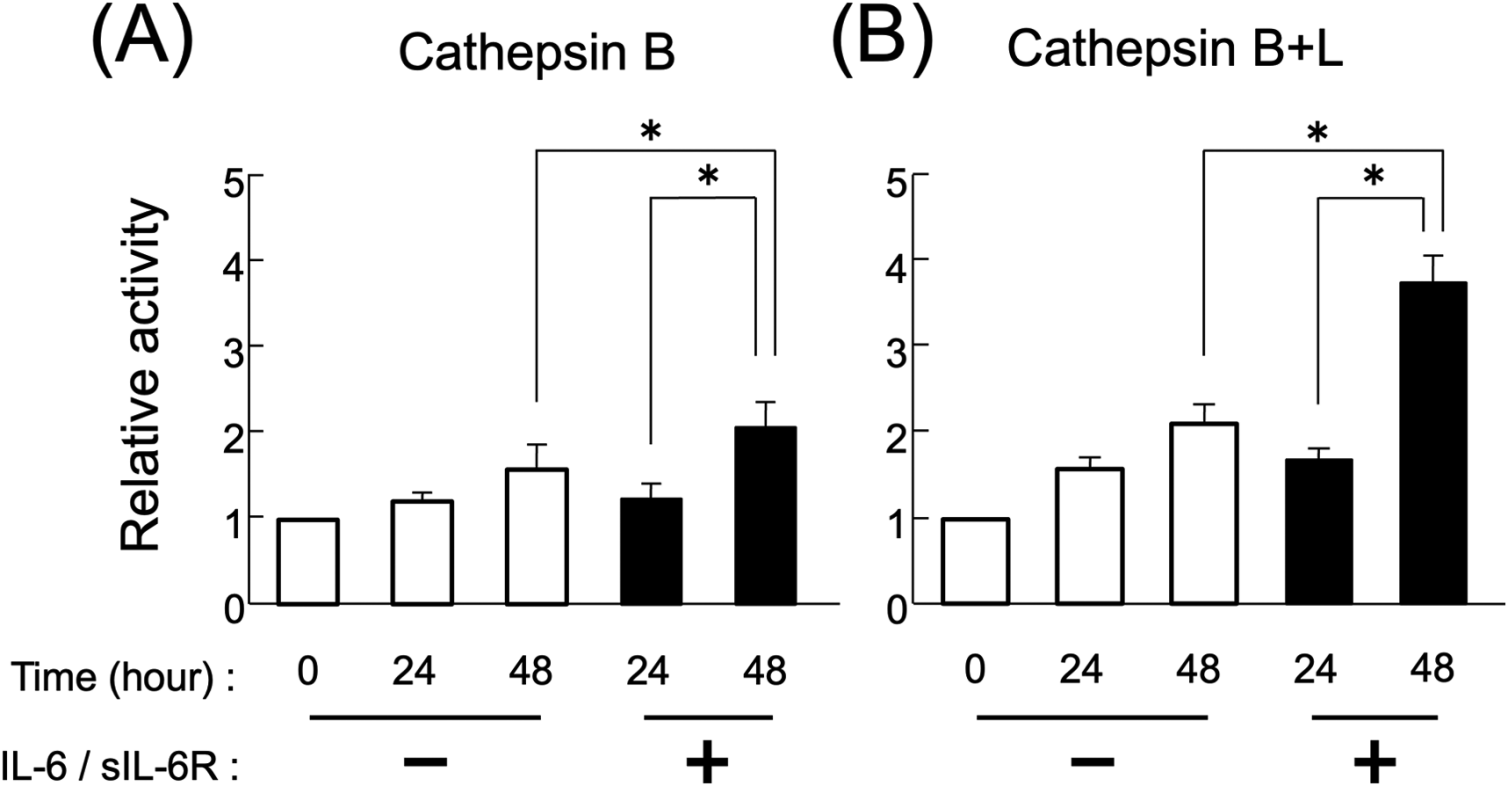
Activity of extracellularly secreted cathepsin B and L precursors following IL-6/sIL-6R complex stimulation. The enzymatic activity levels of cathepsins B and L in the culture supernatants of HGFs were measured at 0, 24, and 48 h of cell culture, both with and without IL-6/sIL-6R complex stimulation. Enzymatic activity was assessed by detecting the amount of fluorescent peptides released from the MCA substrate. **(A)** Cathepsin B, **(B)** cathepsin B+L. The chart displays the culture duration with or without IL-6/sIL-6R complex stimulation on the *x*-axis and the relative activity of the secreted cathepsin B precursors on the *y*-axis. HGFs were sourced from one donor, and three independent experiments were conducted. The error bars in the graph represent the mean ± SD. Statistical analyses were performed using one-way ANOVA and Fisher's PLSD test. *n* = 3; *p < 0.05. ANOVA, analysis of variance; HGFs, human gingival fibroblasts; IL-6, interleukin-6; MCA, 4-methyl-7-coumarylamide; PLSD, protected least significant difference; SD, standard deviation; sIL-6R, soluble interleukin-6 receptor.

Secretion increased following stimulation with the IL-6/sIL-6R complex (Figure 1). At 48 h after IL-6/sIL-6R complex stimulation, the activity of secreted cathepsin B and cathepsin B + L precursors in the sodium acetate solution (pH 5.5) was significantly higher compared with that in the non-stimulation group (Figure 3; *p* < 0.05). In addition, the activity levels of cathepsin B and cathepsin B + L precursors were significantly elevated between 24 and 48 h after IL-6/sIL-6R complex stimulation (Figure 3; *p* < 0.05).

### 3.3 Cav-1 effects on the secretion of cathepsin B and L precursors

**Figure 4:**
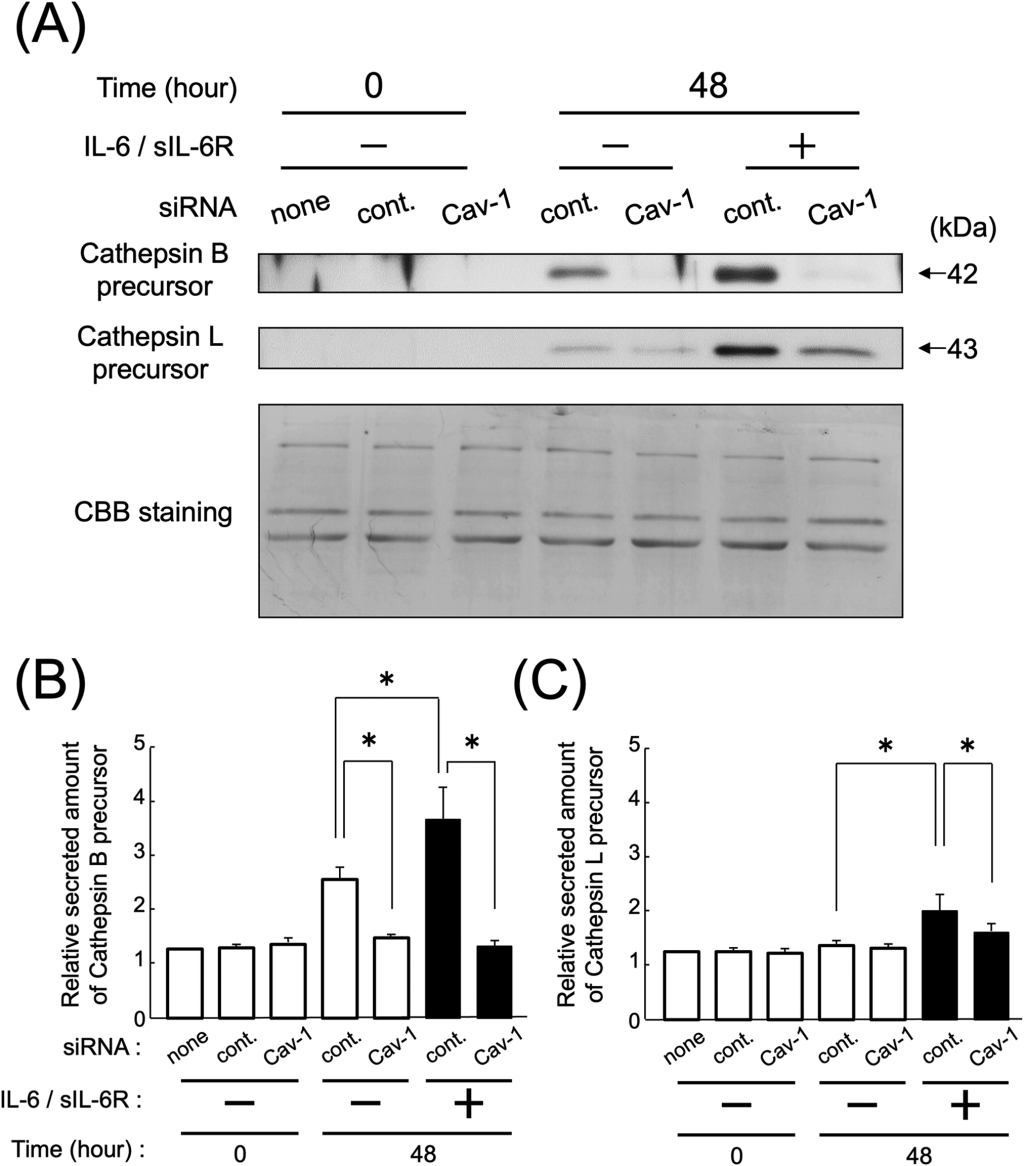
Effect of the knockdown of Cav-1 gene expression on the secretion of cathepsin B and L precursors following IL-6/sIL-6R complex stimulation. The secretion levels of cathepsins B and L in the culture supernatants of HGFs were measured using western blotting. The experiment was conducted on HGFs transfected with either negative control siRNA or Cav-1 siRNA, with or without IL-6/sIL-6R complex stimulation, at 0 and 48 h after cell culture. HGFs were sourced from one donor, and three independent experiments were conducted. The intensity of the bands detected by western blotting was measured using the ImageJ® software, with values calculated relative to the 0 h time point without IL-6/sIL-6R complex stimulation. The error bars in the graph represent the mean ± SD. Statistical analyses were performed using one-way ANOVA and Fisher's PLSD test. **(A)** Western blot images of cathepsin B and L precursors in the cell culture supernatant, with representative images displayed. CBB staining verified that protein amounts loaded in each lane were comparable. **(B)** Densitometric analysis of secreted amounts of cathepsin B precursors. The *y*-axis displays the relative densitometric values of the secreted cathepsin B precursors, whereas the *x*-axis shows the culture duration. The cells were divided into three groups: none (no transfection), control (negative control group transfected with scrambled siRNA), and Cav-1 (knockdown group of Cav-1 gene expression). The effects of IL-6/IL-6R complex stimulation are also shown. *n* = 3; *p < 0.05. **(C)** Densitometric analysis of secreted amounts of cathepsin L precursors. The *y*-axis displays the relative densitometric values of the secreted cathepsin L precursors, whereas the *x*-axis shows the culture duration. The cells were divided into three groups: none (no transfection), control (negative control group transfected with scrambled siRNA), and Cav-1 (knockdown group of Cav-1 gene expression). The effects of IL-6/IL-6R complex stimulation are also shown. *n* = 3; *p < 0.05. ANOVA, analysis of variance; Cav-1, caveolin-1; HGFs, human gingival fibroblasts; IL-6, interleukin-6; PLSD, protected least significant difference; SD, standard deviation; sIL-6R, soluble interleukin-6 receptor; siRNA, small interfering RNA.

The results showed that knocking down Cav-1 gene expression in HGFs using siRNA led to a reduction in the secretion of cathepsin B precursors compared with control siRNA, even in the absence of IL-6/sIL-6R complex stimulation (Figures 4A,B; *p* < 0.05). However, the secretion of cathepsin L precursor was reduced only in the presence of IL-6/sIL-6R complex stimulation (Figures 4A,C; *p* < 0.05). Furthermore, the study demonstrated that the secretion of cathepsin B and L precursors increased in HGFs in which Cav-1 gene expression was knocked down with control siRNA after 48 h of IL-6/sIL-6R complex stimulation (Figure 4; *p* < 0.05).

### 3.4 Suppression of ERK1/2 and JNK signaling pathway effects on the secretion of cathepsins B and L

**Figure 5:**
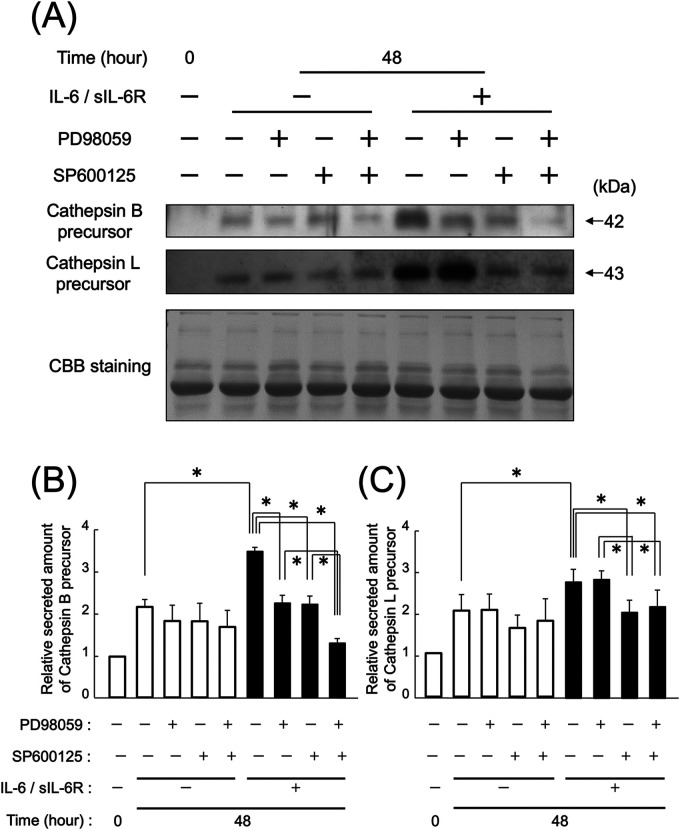
Effect of ERK1/2 and JNK inhibition on the secretion of cathepsin B and L precursors following IL-6/sIL-6R complex stimulation. The impact of the MAPK pathway on the secretion of cathepsin B and L precursors from HGFs with inhibited ERK1/2 and JNK signaling was assessed using western blotting at 0 and 48 h after cell culture, with or without IL-6/sIL-6R complex stimulation. ERK1/2 signaling was inhibited using PD985059, whereas JNK signaling was inhibited using SP600125. HGFs were sourced from two donors, and three independent experiments were conducted. The intensity of the bands detected by western blotting was measured using the ImageJ® software, with values calculated relative to the 0 h time point without IL-6/sIL-6R complex stimulation. The error bars in the graph represent the mean ± SD. Statistical analyses were performed using one-way ANOVA and Fisher's PLSD test. **(A)** Western blot images of cathepsin B and L precursors in the cell culture supernatant, with representative images displayed. CBB staining verified that protein amounts loaded in each lane were comparable. **(B)** Densitometric analysis of secreted amounts of cathepsin B precursors. The *y*-axis displays the relative densitometric values of the secreted cathepsin B precursors, whereas the *x*-axis shows the culture duration with or without IL-6/sIL-6R complex stimulation and inhibitory reagents. *n* = 3; *p < 0.05. **(C)** Densitometric analysis of secreted amounts of cathepsin L precursors. The *y*-axis displays the relative densitometric values of the secreted cathepsin L precursors, whereas the *x*-axis shows the culture duration with or without IL-6/sIL-6R complex stimulation and inhibitory reagents. *n* = 3; *p < 0.05. ANOVA, analysis of variance; CBB, Coomassie Brilliant Blue; ERK, extracellular signal-regulated kinase; HGFs, human gingival fibroblasts; IL-6, interleukin-6; JNK, c-Jun N-terminal kinase; MAPK, mitogen-activated protein kinase; PLSD, protected least significant difference; SD, standard deviation; sIL-6R, soluble interleukin-6 receptor.

At 48 h after IL-6/sIL-6R complex stimulation, the secretion of cathepsin B precursors from HGFs with inhibited ERK1/2 or JNK signaling pathways was reduced compared with that in the uninhibited group (Figures 5A,B; *p* < 0.05). The reduction in secretion of cathepsin B precursors was more pronounced in HGFs with both ERK1/2 and JNK inhibition compared with those with individual pathway inhibition (Figures 5A,B; *p* < 0.05). However, the secretion of cathepsin L precursor from HGFs with ERK1/2 inhibition remained unchanged compared with that in the uninhibited group, whereas the secretion from HGFs with JNK inhibition decreased (Figures 5A,C; *p* < 0.05). In contrast, the secretion of both cathepsin B and L precursors from uninhibited HGFs increased 48 h after IL-6/sIL-6R complex stimulation (Figure 5; *p* < 0.05).

## 4 Discussion

This study found that cathepsin B and L precursors are present outside HGFs. In a previous study, we reported that cathepsin B and L precursors are present inside HGFs (25). Stimulation with the IL-6/sIL-6R complex significantly increased the extracellular secretion of these cathepsin precursors (Figures 1, 2). Furthermore, cathepsin B and L precursors exhibited enzymatic activity and were found in their non-precursor forms (Figure 3). This observation is consistent with previous reports that active cathepsin B precursors secreted from malignant tumor cells can degrade the extracellular matrix (37, 38). In addition, cathepsins B and L in the synovial fluid of patients with rheumatoid arthritis have been shown to degrade type I collagen (39), suggesting that secreted cathepsin B and L precursors in periodontal inflammatory lesions may contribute to the degradation of extracellular matrix components, such as collagen, in periodontal tissue. Based on these findings, we hypothesized that regulating the secretion of cathepsin B and L from HGFs in periodontal inflammatory lesions could be crucial for managing the pathogenesis of periodontitis. Therefore, this study aimed to investigate the extracellular secretory mechanisms of cathepsin B and L precursors following IL-6/sIL-6R complex stimulation to elucidate differences in intracellular cathepsin production mechanisms.

**Figure 6:**
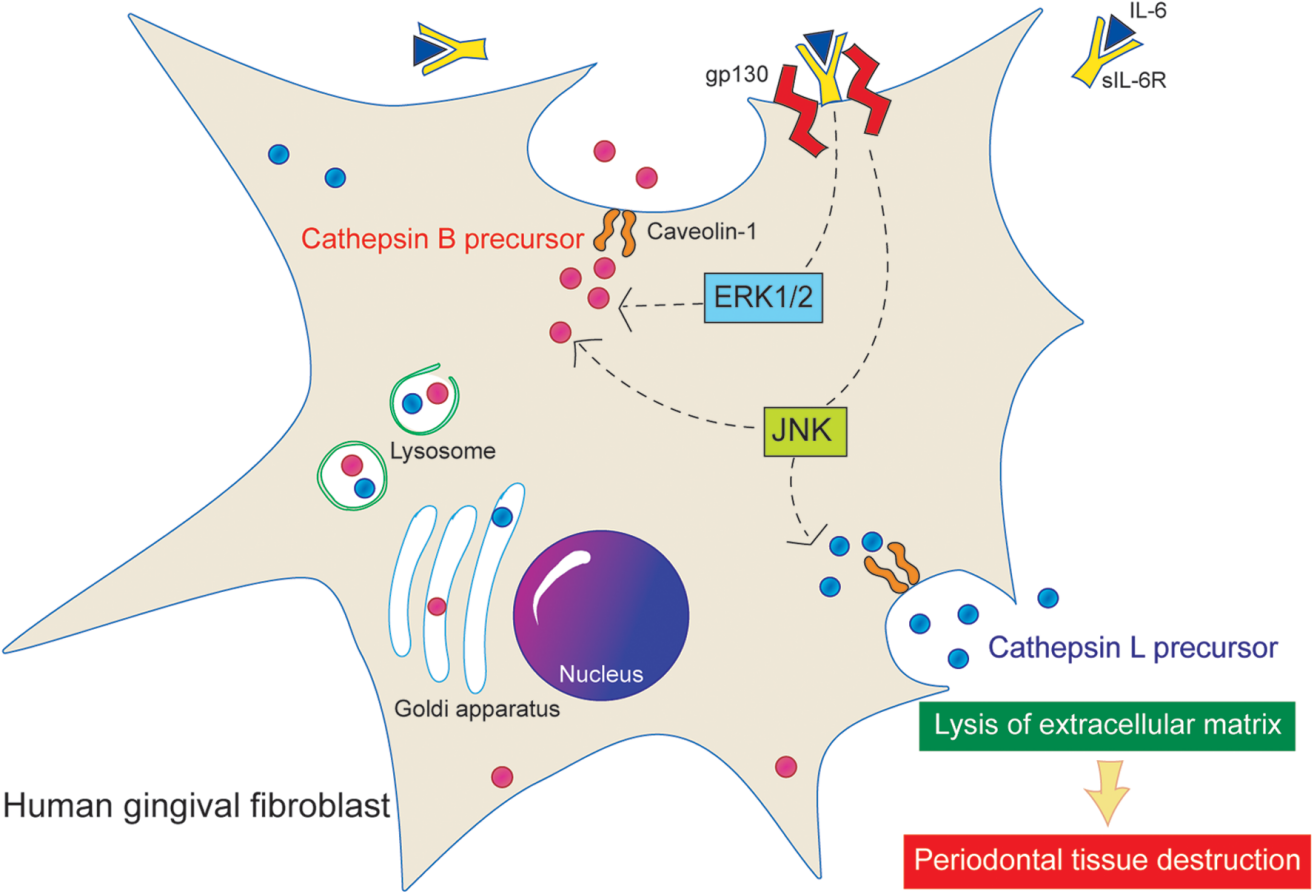
Proposed signaling pathway of cathepsin B and L. Stimulation of the IL-6/sIL-6R complex increases the extracellular secretion of active precursors of cathepsins B and L in HGFs. In addition, Cav-1 and ERK1/2 are implicated in the secretion of cathepsin B precursors.

The membrane protein Cav-1 plays a crucial role in enhancing the production of cathepsins B and L in HGFs following IL-6 stimulation (25). This study elucidated that the IL-6/sIL-6R complex enhances the secretion of cathepsins B and L via the Cav-1 and ERK1/2 pathways (Figure 6). It demonstrated that knockdown of Cav-1 gene expression using siRNA significantly reduced the secretion of cathepsin B and L precursors from HGFs upon IL-6/sIL-6R stimulation. Notably, cathepsin B secretion was diminished even in the absence of IL-6/sIL-6R stimulation (Figures 1, 4), suggesting that Cav-1 is essential not only for IL-6-induced signaling but also for the extracellular transport of cathepsin B. This suggests that Cav-1 is involved not only in the IL-6-induced signaling pathways that stimulate cathepsin B production but also in the extracellular transport of cathepsin B from intracellular membranes. Supporting this hypothesis, breast cancer cells co-expressing Cav-1 and cathepsin B exhibit higher malignancy than other cells owing to their increased invasion and metastasis (40), which is likely attributed to increased secretion of cathepsin B and subsequent extracellular matrix degradation. Similarly, a previous study indicated that suppressing Cav-1 gene expression reduces the production and secretion of cathepsin B precursors in colon cancer cells (41). In addition, Cav-1 is associated with the production of other extracellular matrix-degrading proteins, such as MMPs (42). This study underscores the critical role of Cav-1 in destroying periodontal tissue through its interaction with cathepsins.

The mitogen-activated protein kinase (MAPK) signaling pathway is an inflammatory cascade triggered by IL-6. Previous research has shown that within the MAPK pathway, only JNK, not ERK1/2, is involved in the intracellular production of cathepsins B and L in HGFs stimulated by IL-6 (27). Another report has shown that the expression of intracellular cathepsin S in human ﬁbroblast-like periodontal ligament cells has been upregulated under stimulation with IL-1β and periodontopathic *Fusobacterium nucleatum* (43). In this study, we explored the relationship between MAPK signaling and the extracellular secretion of cathepsins B and L. Our findings indicate that inhibiting either JNK or ERK1/2 significantly reduced the secretion of cathepsin B, whereas only JNK inhibition led to a decrease in the secretion of cathepsin L. These results suggest that cathepsins B and L may be regulated by distinct secretion mechanisms, with ERK1/2 specifically involved in the secretion of cathepsin B (Figures 5, 6).

Although the mechanisms underlying the extracellular secretion of cathepsins remain unclear, existing literature suggests that annexin II-a protein that binds to phospholipids and membranes and is involved in exocytosis–plays a role in the secretion of cathepsin B via Cav-1 in umbilical vein endothelial cells (44, 45). Therefore, ERK1/2 may be associated with the production and function of proteins, such as annexin II, which facilitate the extracellular transport of cathepsin B. Future research should focus on elucidating the exocytosis mechanisms of cathepsin B by identifying the specific proteins involved in its extracellular transport and their interactions with Cav-1.

Cathepsins are crucial proteases for breaking down the extracellular matrix into amino acids. However, in inflamed tissues, they can be found extracellularly and may contribute to tissue destruction. This study demonstrated that HGFs secrete cathepsin B precursors and suggested that cathepsin B and L precursors may be active in chronic inflammatory tissues. Further research is needed to enhance our understanding of how to inhibit the contribution of secreted cathepsins to periodontal tissue destruction without disrupting the function of intracellular cathepsins necessary for homeostasis. Such insights could inform new strategies to mitigate IL-6-related chronic inflammation and its effects.

## 5 Conclusions

Stimulation of the IL-6/sIL-6R complex increases the extracellular secretion of active precursors of cathepsins B and L in HGFs. In addition, Cav-1 and ERK1/2 are implicated in the secretion of cathepsin B precursors.

## Data Availability

The original contributions presented in the study are included in the article/Supplementary Material, further inquiries can be directed to the corresponding author.
