# Supplementary figures and images for: Interleukin-6/soluble IL-6 receptor-induced secretion of cathepsin B and L from human gingival fibroblasts is regulated by caveolin-1 and ERK1/2 pathways
Source: Front Dent Med. 2025 Mar 11;6:1547222. doi: 10.3389/fdmed.2025.1547222 (PMC11933118; doi:10.3389/fdmed.2025.1547222)

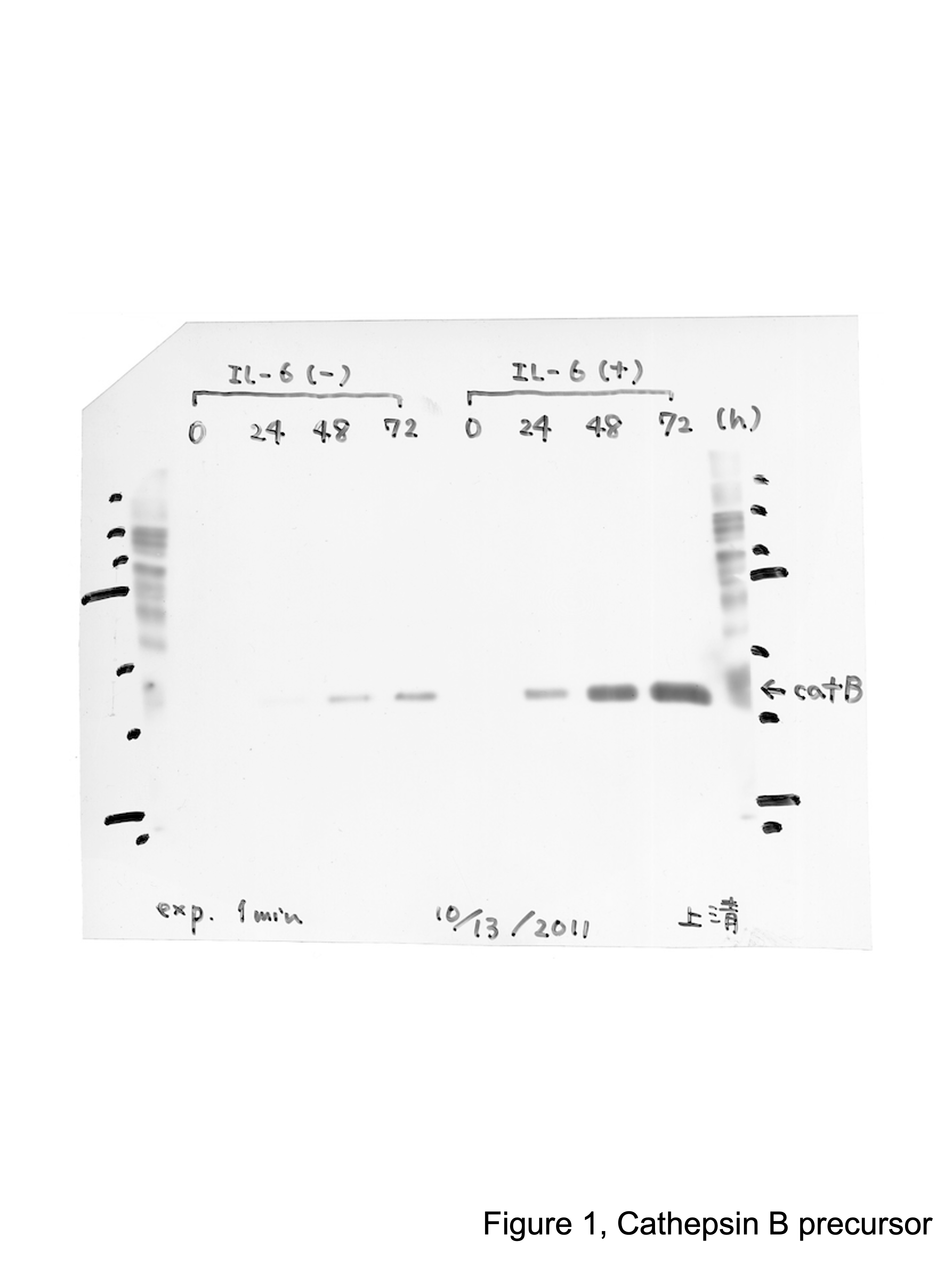

Supplement: Supplementary file 1 [file Image1.tiff]

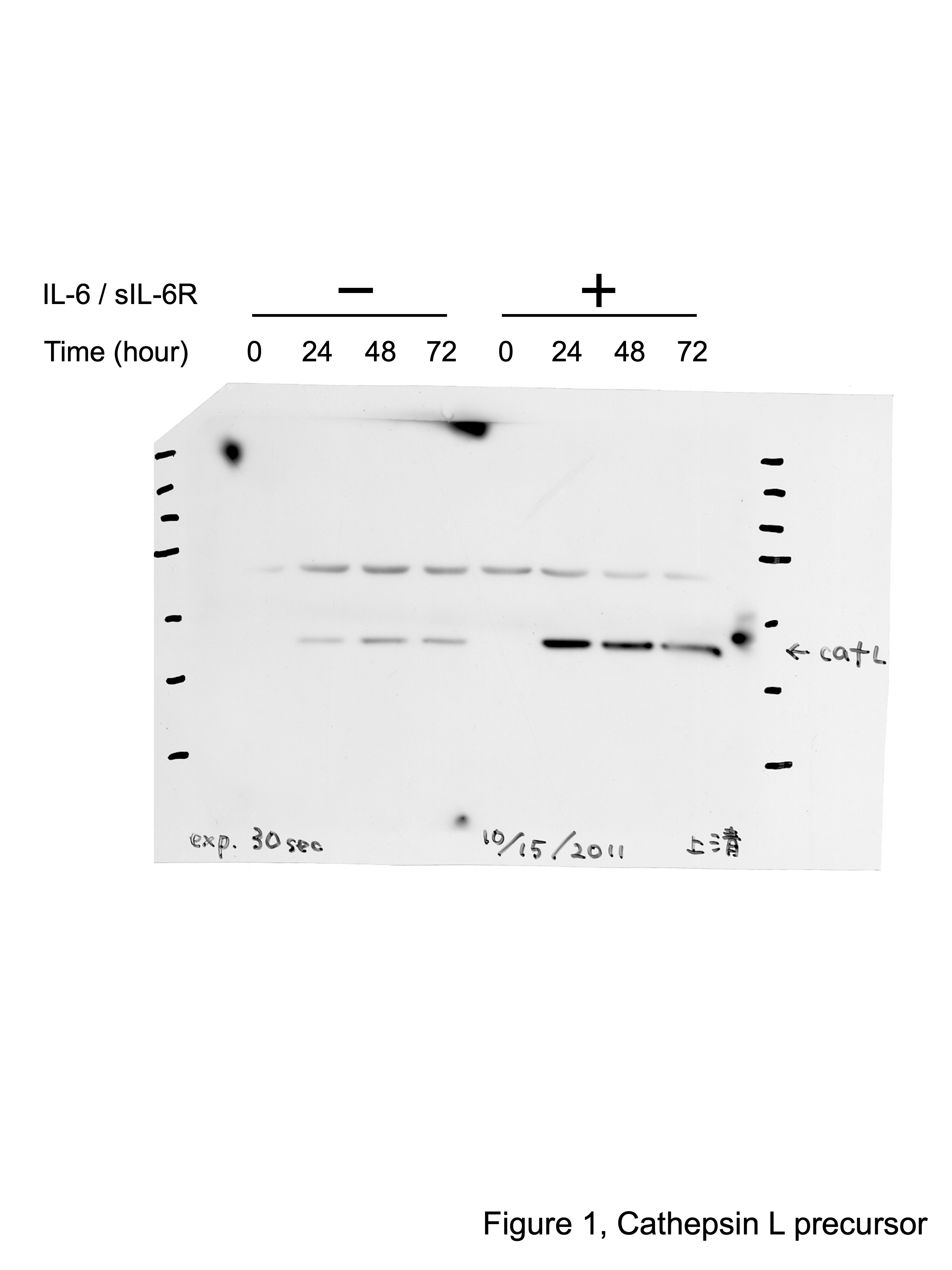

Supplement: Supplementary file 2 [file Image2.tiff]

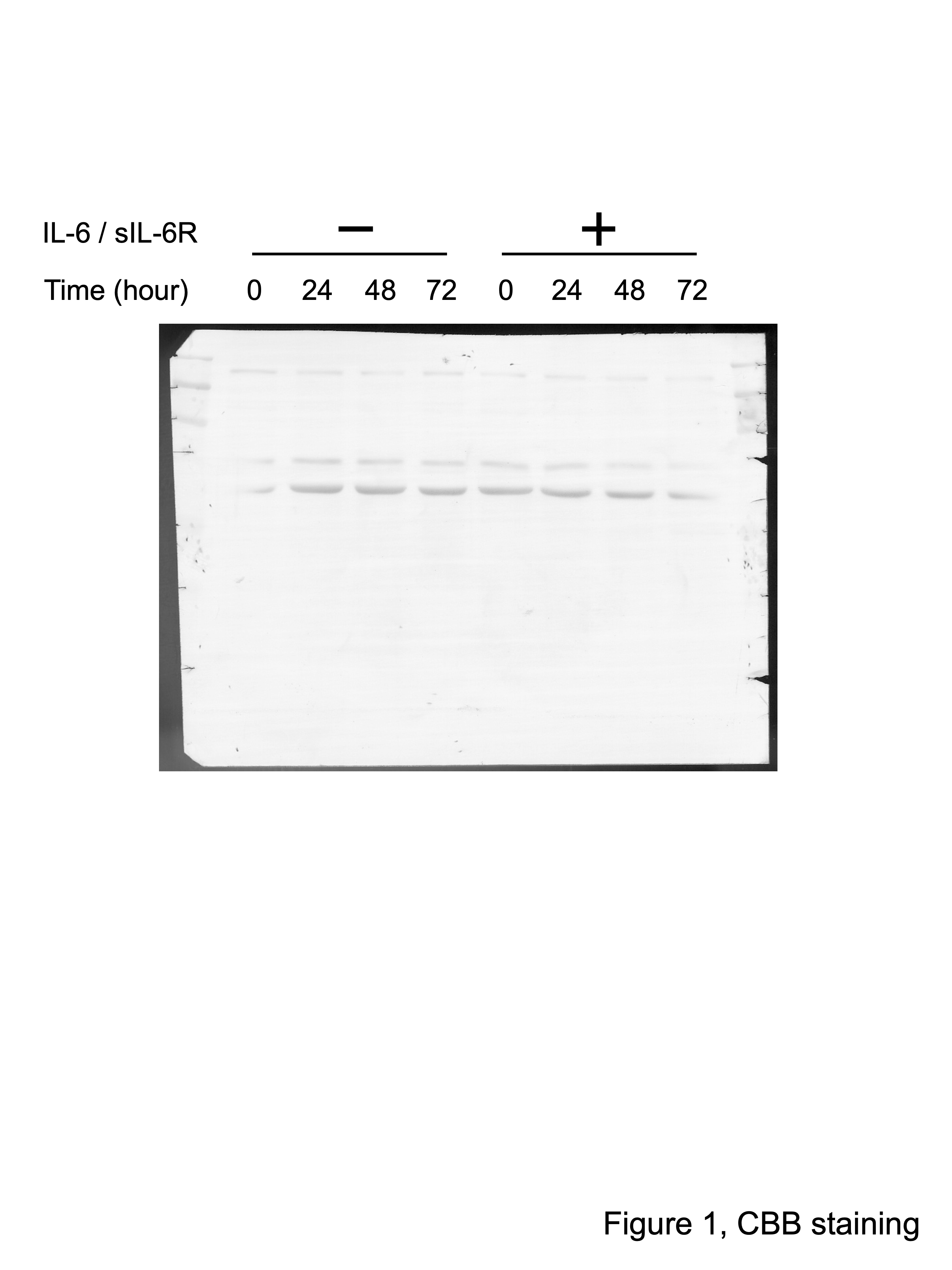

Supplement: Supplementary file 3 [file Image3.tiff]

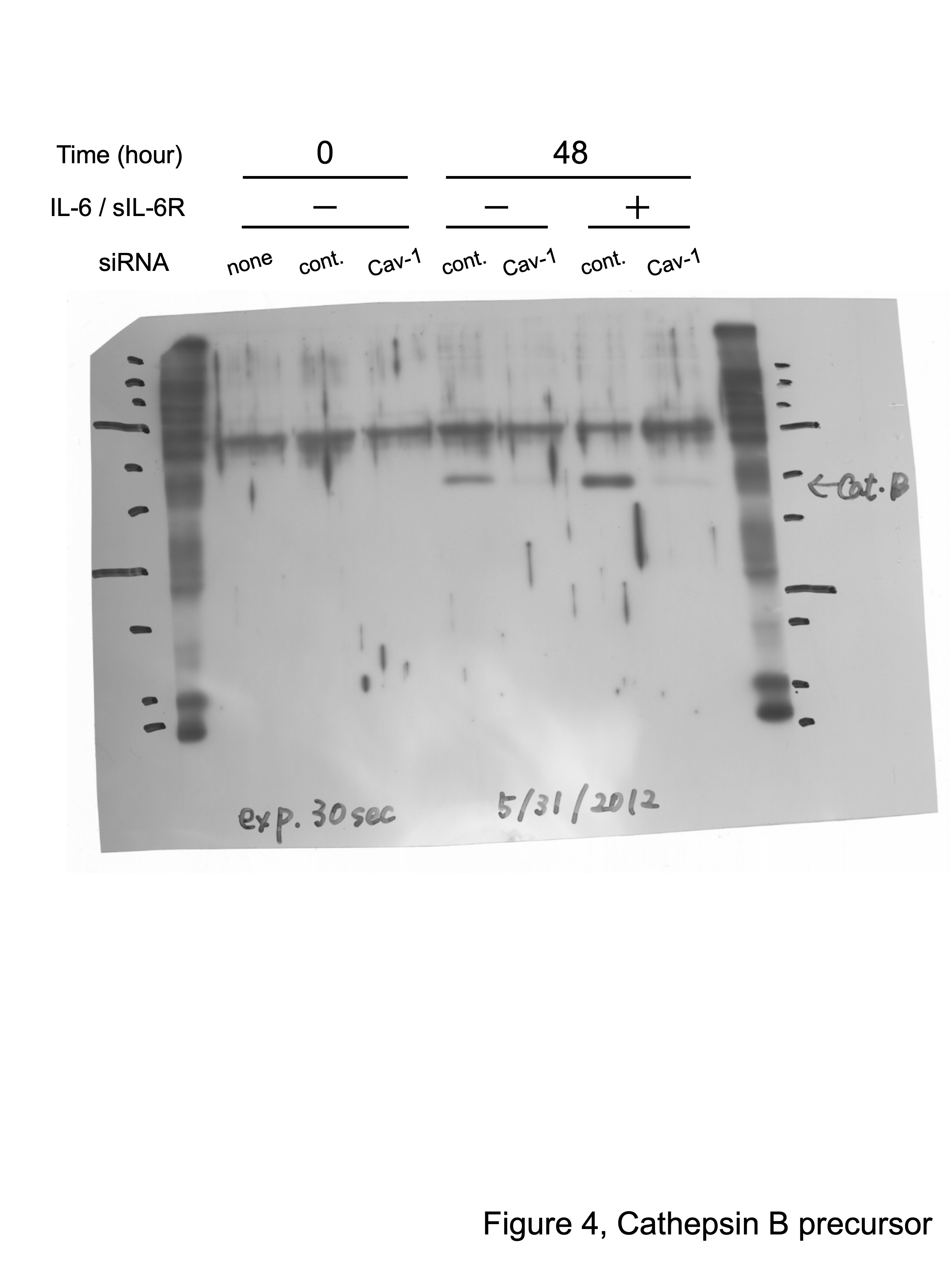

Supplement: Supplementary file 4 [file Image4.tiff]

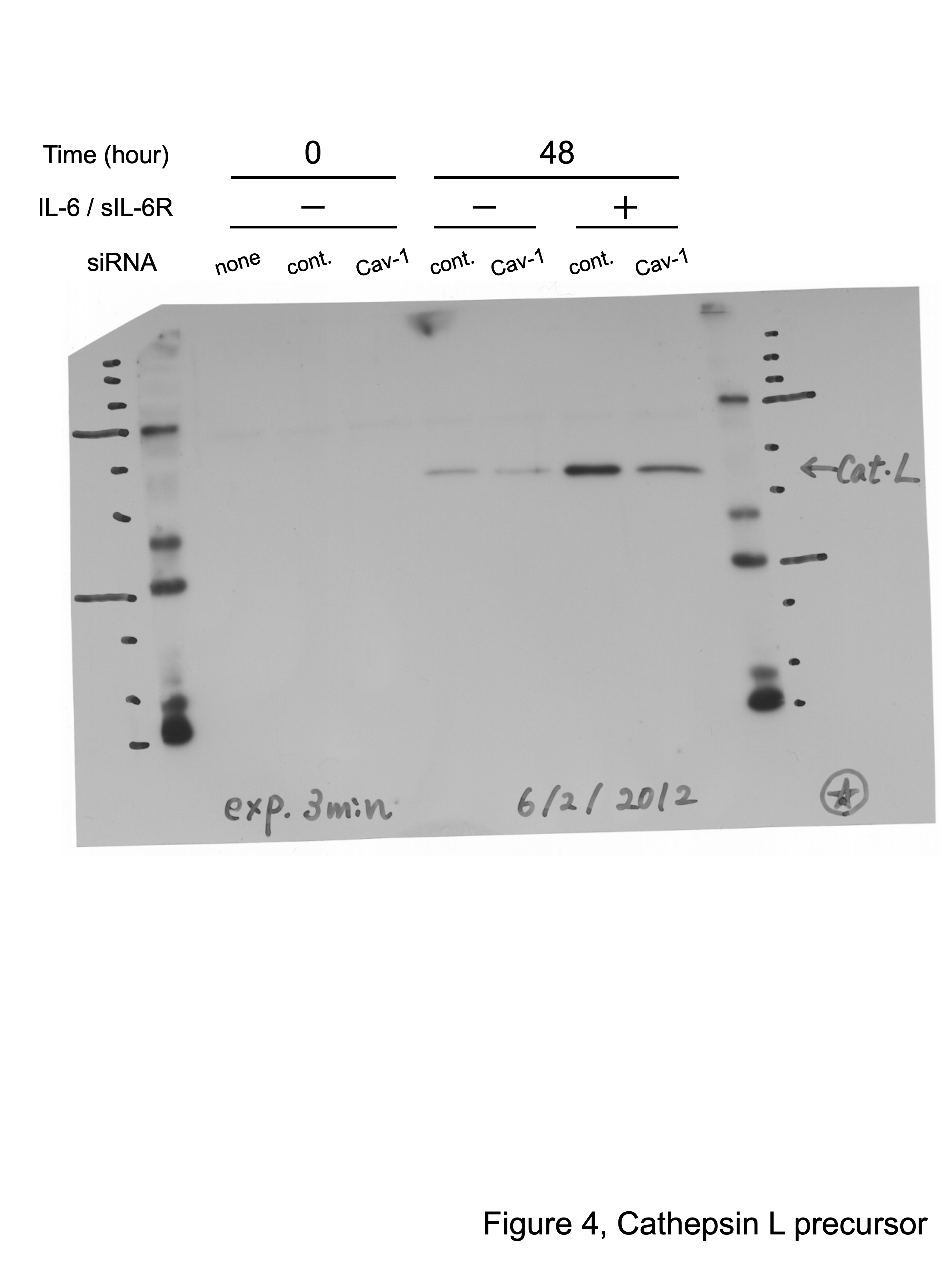

Supplement: Supplementary file 5 [file Image5.tiff]

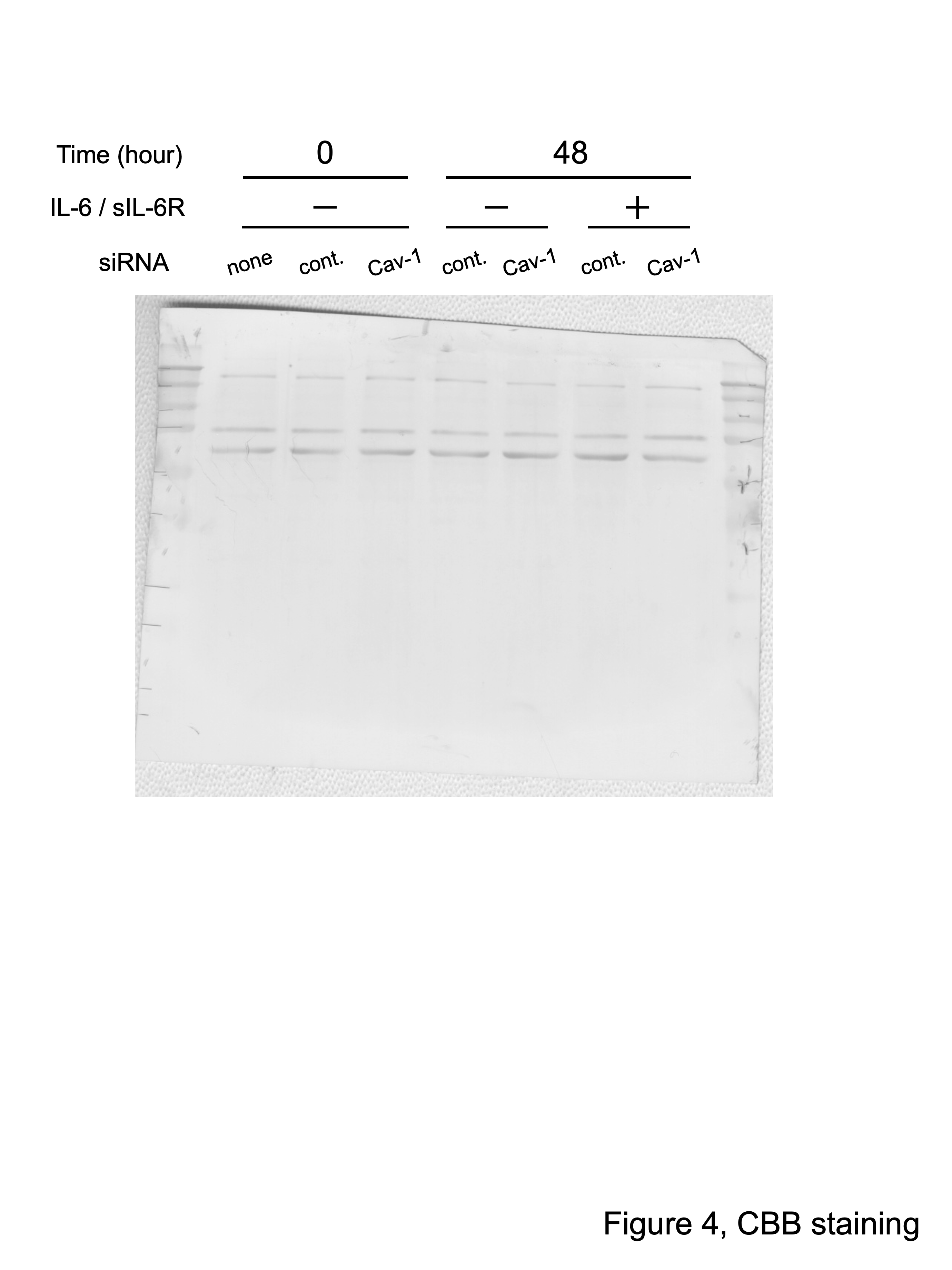

Supplement: Supplementary file 6 [file Image6.tiff]

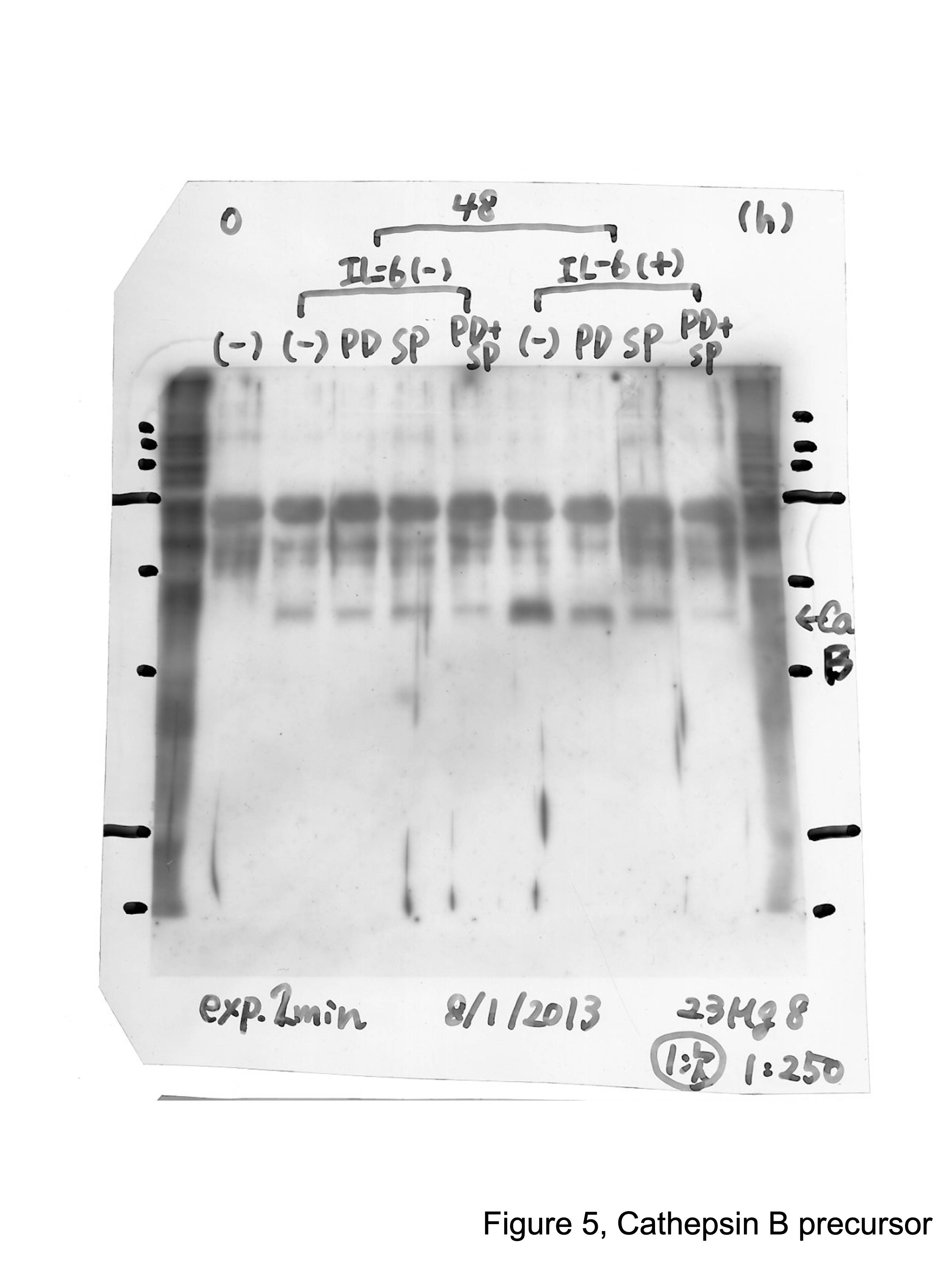

Supplement: Supplementary file 7 [file Image7.tiff]

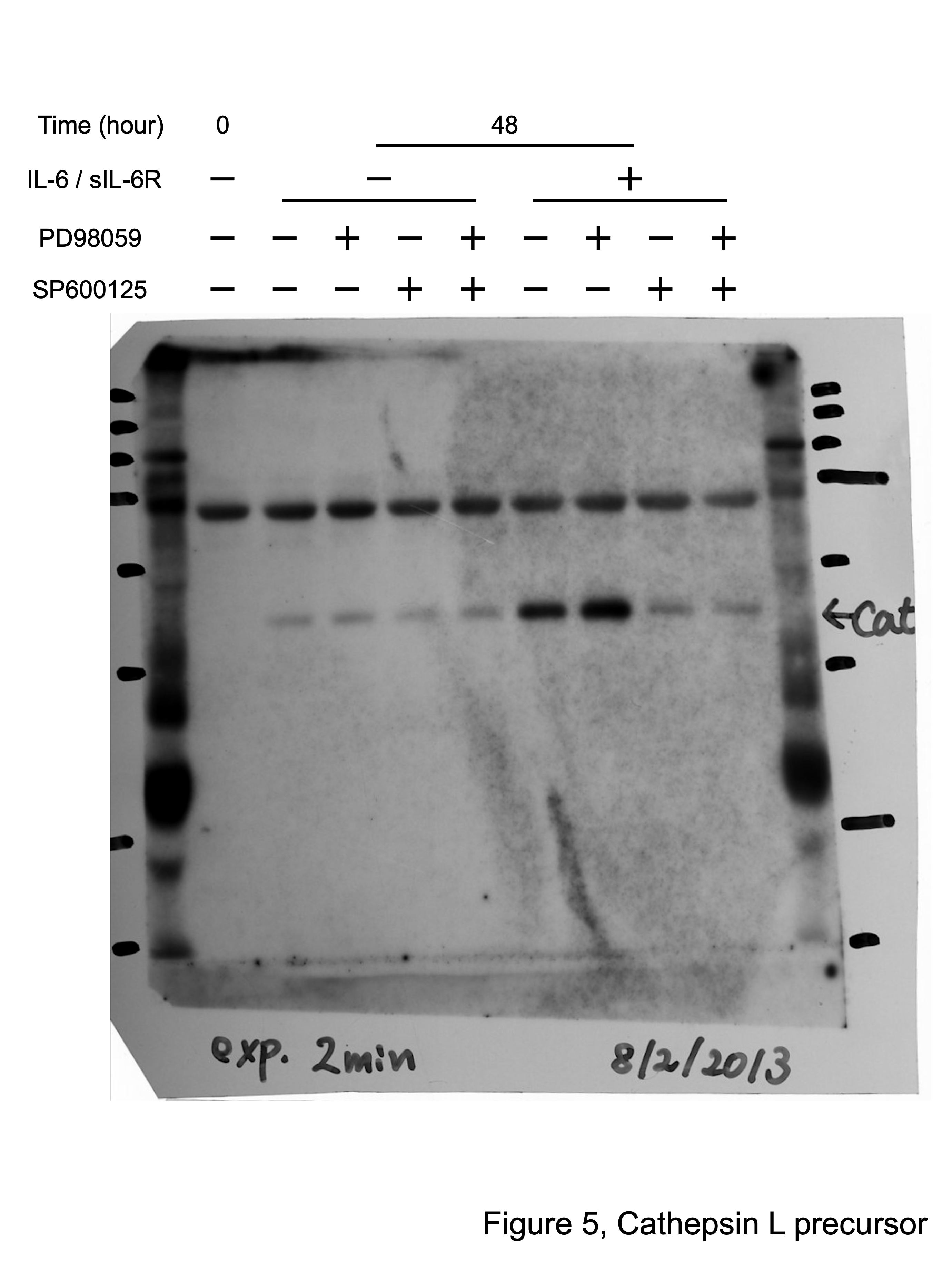

Supplement: Supplementary file 8 [file Image8.tiff]

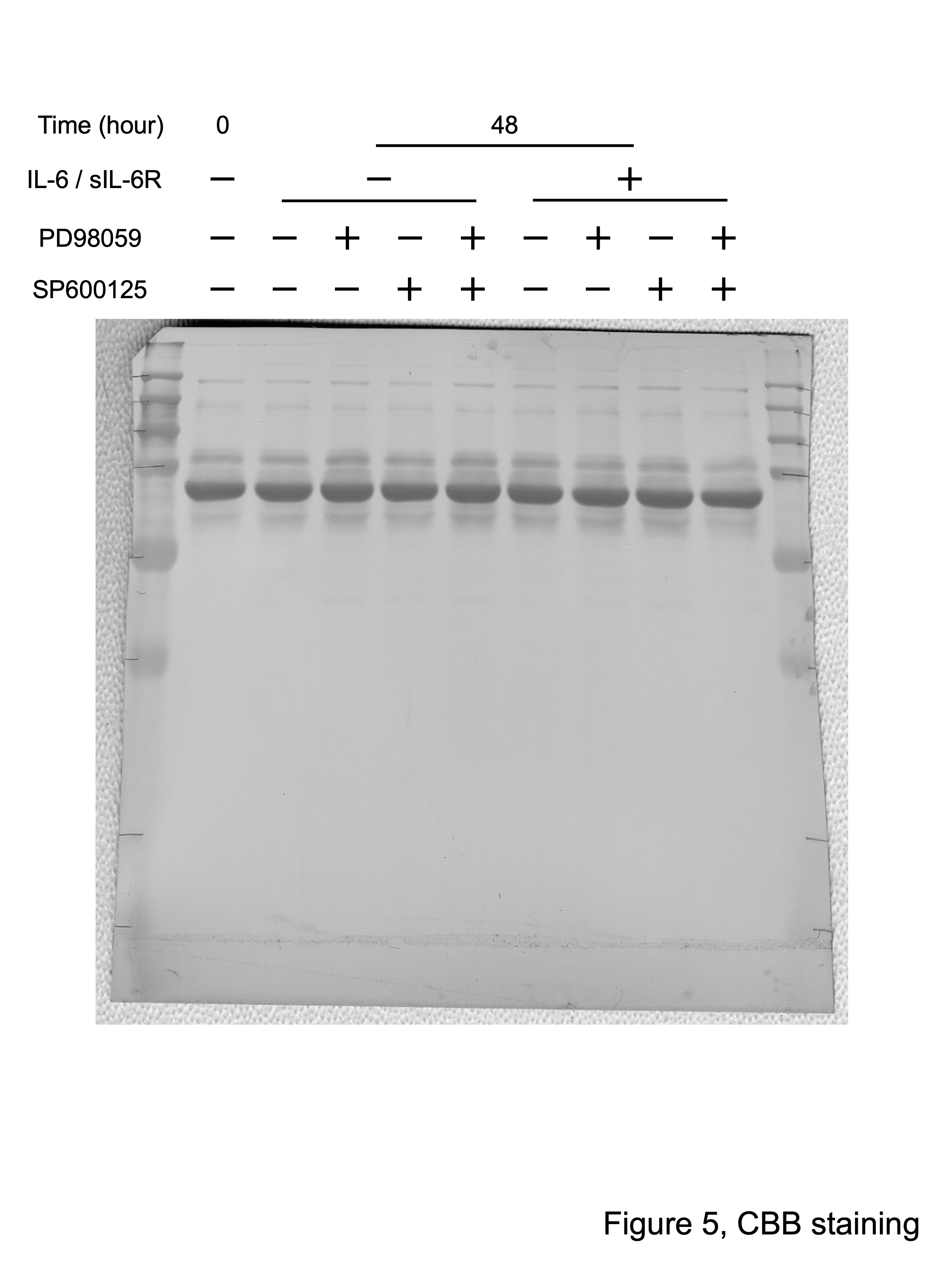

Supplement: Supplementary file 9 [file Image9.tiff]
